# *catena*-Poly[2-methyl-1*H*-imidazol-3-ium [(aqua­chloridocadmate)-di-μ-chlorido]]

**DOI:** 10.1107/S1600536811042905

**Published:** 2011-10-22

**Authors:** Run-Qiang Zhu

**Affiliations:** a Ordered Matter Science Research Center, College of Chemistry and Chemical Engineering, Southeast University, Nanjing 211189, People’s Republic of China

## Abstract

The asymmetric unit of the title compound, {(C_4_H_7_N_2_)[CdCl_3_(H_2_O)]}_*n*_, contains one 1-methyl-1*H*-imidazol-3-ium cation, one Cd^II^ atom, three Cl atoms and one water mol­ecule. Adjacent Cd ions are inter­connected alternately by paired Cl^−^ bridges to generate an infinite one-dimensional coordination chain along the *b* axis. In the chain, the crystallographically unique Cd^II^ atom, with a distorted octa­hedral geometry, is coordinated by five Cl^−^ ions and one water mol­ecule. Intra-chain O—H⋯Cl hydrogen bonding and N—H⋯Cl hydrogen bonding between the cations and the anionic chains consolidate the crystal packing.

## Related literature

For general background to ferroelectric metal-organic compounds with framework structures, see: Fu *et al.* (2009 ▶); Ye *et al.* (2006 ▶); Zhang *et al.* (2008 ▶, 2010 ▶).
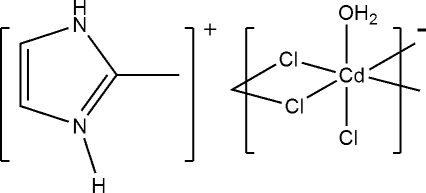


## Experimental

### 

#### Crystal data

(C_4_H_7_N_2_)[CdCl_3_(H_2_O)]
*M* *_r_* = 319.88
Monoclinic, 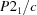

*a* = 9.0479 (18) Å
*b* = 14.922 (3) Å
*c* = 7.4711 (15) Å
β = 94.17 (3)°
*V* = 1006.0 (3) Å^3^
*Z* = 4
Mo *K*α radiation
μ = 2.92 mm^−1^
*T* = 293 K
0.30 × 0.25 × 0.20 mm

#### Data collection

Rigaku SCXmini diffractometer
Absorption correction: multi-scan (*CrystalClear*; Rigaku, 2005 ▶) *T*_min_ = 0.421, *T*_max_ = 0.558
10317 measured reflections
2308 independent reflections
2038 reflections with *I* > 2σ(*I*)
*R*_int_ = 0.082

#### Refinement

*R*[*F* ^2^ > 2σ(*F* ^2^)] = 0.033
*wR*(*F* ^2^) = 0.083
*S* = 1.12
2308 reflections
102 parameters
H-atom parameters constrained
Δρ_max_ = 0.72 e Å^−3^
Δρ_min_ = −0.71 e Å^−3^

### 

Data collection: *CrystalClear* (Rigaku, 2005 ▶); cell refinement: *CrystalClear*; data reduction: *CrystalClear*; program(s) used to solve structure: *SHELXS97* (Sheldrick, 2008 ▶); program(s) used to refine structure: *SHELXL97* (Sheldrick, 2008 ▶); molecular graphics: *DIAMOND* (Brandenburg & Putz, 2005 ▶); software used to prepare material for publication: *SHELXL97*.

## Supplementary Material

Crystal structure: contains datablock(s) I, global. DOI: 10.1107/S1600536811042905/vm2126sup1.cif

Structure factors: contains datablock(s) I. DOI: 10.1107/S1600536811042905/vm2126Isup2.hkl

Additional supplementary materials:  crystallographic information; 3D view; checkCIF report

## Figures and Tables

**Table 1:** Hydrogen-bond geometry (Å, °)

*D*—H⋯*A*	*D*—H	H⋯*A*	*D*⋯*A*	*D*—H⋯*A*
N2—H2*A*⋯O1	0.86	2.13	2.884 (3)	146
N1—H1*D*⋯Cl1^i^	0.86	2.33	3.163 (3)	164
O1—H1*G*⋯Cl1^ii^	0.85	2.40	3.250 (2)	174
O1—H1*F*⋯Cl1^iii^	0.85	2.44	3.174 (2)	146

Symmetry codes: (i) 
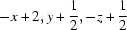
; (ii) 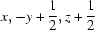; (iii) 
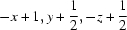
.

**Fig. 1.**
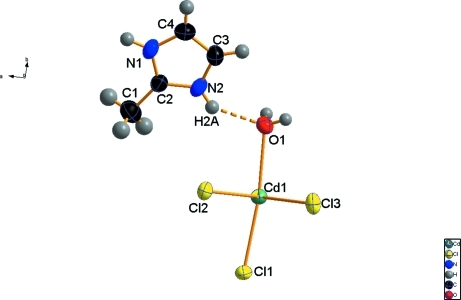
A partial packing diagram of the title compound, with the displacement ellipsoids drawn at the 30% probability level, the intramolecular hydrogen bond is shown as a dashed line.

**Fig. 2.**
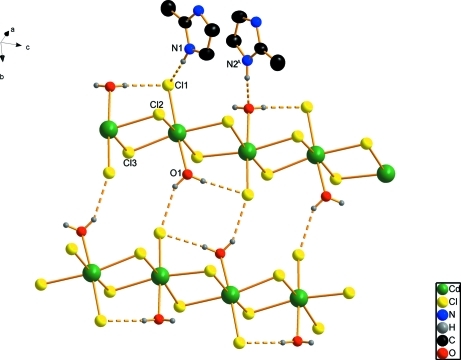
Packing diagram of the title compound, hydrogen bonds are shown as dashed lines.

## supplementary crystallographic information

### Comment

The basic method to find potential ferroelectric phase change materials is the measurement of the dielectric constant as function of temperature (Fu *et al.*, 2009; Ye *et al.*, 2006; Zhang *et al.*, 2008, 2010). Unfortunately, the title compound's dielectric constant does not change from 80 K to 298 K (m.p. 319–329).

X-ray analysis (Fig. 1) revealed that the title compound possesses 1-D chain structures. In the chain, the Cd atoms are connected by two Cl atoms acting as bridges between Cd1 and Cd1[*x*, 0.5 - *y*, 1/2 + *z*] centers. The Cd—Cl(µ_2_) distances from 2.5973 (11) to 2.6293 (12) Å are slightly longer than that of Cd—Cl(terminal) 2.5916 (10) Å. It is interesting to note that the free 2-methyl imidazole molecules extend the 1-D host chains into a 3-D supramolecular network *via* the hydrogen-bonded interactions (Table 1, Fig. 2).

### Experimental

A mixture of 2-methyl imidazole (2.4 g, 30 mmol), cadmium chloride (3.15 g, 10 mmol) in water was stirred for several days at ambient temperature. Colourless block crystals were obtained.

### Refinement

All H atoms were placed in geometrically idealized positions and constrained to ride on their parent atoms with aromatic C—H = 0.93 Å and methyl C—H = 0.96 Å and N—H = 0.86, with *U*_iso_(H) = 1.2*U_eq_*(C,N)and 1.5*U*_eq_(C) for methyl H atoms. The H atoms of the water molecule were restrained with O—H = 0.85 Å yielding O1—H1G = 0.8501 Å and O1—H1F = 0.8500 Å, with *U*_iso_(H) = 1.2*U*_eq_(O)

### Crystal data

**Table:**

(C_4_H_7_N_2_)[CdCl_3_(H_2_O)]	*F*(000) = 616
*M**_r_* = 319.88	*D*_x_ = 2.112 Mg m^−^^3^
Monoclinic, *P*2_1_/*c*	Mo *K*α radiation, λ = 0.71073 Å
Hall symbol: -P 2ybc	Cell parameters from 2308 reflections
*a* = 9.0479 (18) Å	θ = 2.2–27.5°
*b* = 14.922 (3) Å	µ = 2.92 mm^−^^1^
*c* = 7.4711 (15) Å	*T* = 293 K
β = 94.17 (3)°	Block, colourless
*V* = 1006.0 (3) Å^3^	0.30 × 0.25 × 0.20 mm
*Z* = 4	

### Data collection

**Table:**

Rigaku SCXmini diffractometer	2038 reflections with *I* > 2σ(*I*)
Radiation source: fine-focus sealed tube	*R*_int_ = 0.082
graphite	θ_max_ = 27.5°, θ_min_ = 3.1°
ω scans	*h* = −11→11
Absorption correction: multi-scan (*CrystalClear*; Rigaku, 2005)	*k* = −19→19
*T*_min_ = 0.421, *T*_max_ = 0.558	*l* = −9→9
10317 measured reflections	2 standard reflections every 150 reflections
2308 independent reflections	intensity decay: none

### Refinement

**Table:**

Refinement on *F*^2^	Secondary atom site location: difference Fourier map
Least-squares matrix: full	Hydrogen site location: inferred from neighbouring sites
*R*[*F*^2^ > 2σ(*F*^2^)] = 0.033	H-atom parameters constrained
*wR*(*F*^2^) = 0.083	*w* = 1/[σ^2^(*F*_o_^2^) + (0.0174*P*)^2^ + 0.0243*P*] where *P* = (*F*_o_^2^ + 2*F*_c_^2^)/3
*S* = 1.12	(Δ/σ)_max_ = 0.001
2308 reflections	Δρ_max_ = 0.72 e Å^−^^3^
102 parameters	Δρ_min_ = −0.71 e Å^−^^3^
0 restraints	Extinction correction: *SHELXL97* (Sheldrick, 2008), Fc^*^=kFc[1+0.001xFc^2^λ^3^/sin(2θ)]^-1/4^
Primary atom site location: structure-invariant direct methods	Extinction coefficient: 0.0729 (19)

### Special details

**Table:**

Geometry. All e.s.d.'s (except the e.s.d. in the dihedral angle between two l.s. planes) are estimated using the full covariance matrix. The cell e.s.d.'s are taken into account individually in the estimation of e.s.d.'s in distances, angles and torsion angles; correlations between e.s.d.'s in cell parameters are only used when they are defined by crystal symmetry. An approximate (isotropic) treatment of cell e.s.d.'s is used for estimating e.s.d.'s involving l.s. planes.
Refinement. Refinement of *F*^2^ against ALL reflections. The weighted *R*-factor *wR* and goodness of fit *S* are based on *F*^2^, conventional *R*-factors *R* are based on *F*, with *F* set to zero for negative *F*^2^. The threshold expression of *F*^2^ > σ(*F*^2^) is used only for calculating *R*-factors(gt) *etc*. and is not relevant to the choice of reflections for refinement. *R*-factors based on *F*^2^ are statistically about twice as large as those based on *F*, and *R*-factors based on ALL data will be even larger.

### Fractional atomic coordinates and isotropic or equivalent isotropic displacement parameters (Å^2^)

**Table:**

	*x*	*y*	*z*	*U*_iso_*/*U*_eq_	
Cd1	0.57821 (2)	0.235443 (17)	0.32343 (3)	0.02778 (14)	
Cl3	0.38194 (10)	0.26229 (6)	0.06038 (12)	0.0358 (2)	
Cl2	0.78469 (10)	0.25735 (5)	0.08951 (12)	0.0318 (2)	
Cl1	0.59444 (8)	0.06319 (6)	0.28507 (11)	0.0381 (2)	
N2	0.8602 (3)	0.4748 (2)	0.2374 (4)	0.0461 (8)	
H2A	0.8000	0.4318	0.2563	0.055*	
C2	1.0070 (4)	0.4697 (2)	0.2644 (4)	0.0395 (8)	
C3	0.8185 (4)	0.5580 (3)	0.1755 (5)	0.0487 (10)	
H3	0.7224	0.5780	0.1460	0.058*	
C1	1.0943 (4)	0.3909 (3)	0.3283 (5)	0.0588 (12)	
H1A	1.1219	0.3567	0.2272	0.088*	
H1B	1.0357	0.3544	0.4018	0.088*	
H1C	1.1819	0.4105	0.3975	0.088*	
N1	1.0572 (3)	0.5493 (2)	0.2218 (4)	0.0458 (8)	
H1D	1.1492	0.5643	0.2283	0.055*	
C4	0.9424 (4)	0.6046 (3)	0.1659 (5)	0.0509 (10)	
H4	0.9500	0.6637	0.1282	0.061*	
O1	0.5944 (2)	0.39528 (16)	0.3580 (3)	0.0371 (6)	
H1G	0.5922	0.4101	0.4677	0.045*	
H1F	0.5230	0.4207	0.2978	0.045*	

### Atomic displacement parameters (Å^2^)

**Table:**

	*U*^11^	*U*^22^	*U*^33^	*U*^12^	*U*^13^	*U*^23^
Cd1	0.0307 (2)	0.0277 (2)	0.02493 (19)	−0.00076 (9)	0.00188 (13)	0.00017 (8)
Cl3	0.0272 (5)	0.0527 (6)	0.0275 (5)	0.0054 (3)	0.0023 (4)	0.0021 (3)
Cl2	0.0266 (4)	0.0399 (5)	0.0287 (4)	−0.0026 (3)	0.0010 (4)	0.0007 (3)
Cl1	0.0325 (4)	0.0251 (4)	0.0565 (5)	−0.0026 (3)	0.0024 (4)	−0.0006 (4)
N2	0.0311 (15)	0.044 (2)	0.063 (2)	−0.0122 (14)	0.0037 (15)	0.0024 (15)
C2	0.0347 (19)	0.040 (2)	0.044 (2)	−0.0029 (16)	0.0047 (17)	−0.0045 (16)
C3	0.0349 (19)	0.049 (2)	0.063 (2)	0.0025 (18)	0.0062 (18)	0.002 (2)
C1	0.057 (3)	0.057 (3)	0.062 (3)	0.012 (2)	0.000 (2)	0.0006 (19)
N1	0.0290 (15)	0.049 (2)	0.060 (2)	−0.0095 (14)	0.0037 (14)	−0.0025 (16)
C4	0.052 (2)	0.037 (2)	0.064 (3)	−0.0008 (18)	0.004 (2)	0.0003 (18)
O1	0.0370 (12)	0.0341 (15)	0.0402 (12)	0.0050 (10)	0.0029 (10)	0.0065 (10)

### Geometric parameters (Å, °)

**Table:**

Cd1—O1	2.402 (2)	C2—C1	1.476 (5)
Cd1—Cl3	2.5824 (12)	C3—C4	1.325 (5)
Cd1—Cl1	2.5916 (10)	C3—H3	0.9300
Cd1—Cl3^i^	2.5973 (11)	C1—H1A	0.9600
Cd1—Cl2^i^	2.6293 (12)	C1—H1B	0.9600
Cd1—Cl2	2.6694 (11)	C1—H1C	0.9600
Cl3—Cd1^ii^	2.5973 (11)	N1—C4	1.368 (4)
Cl2—Cd1^ii^	2.6293 (12)	N1—H1D	0.8600
N2—C2	1.331 (4)	C4—H4	0.9300
N2—C3	1.369 (5)	O1—H1G	0.8501
N2—H2A	0.8600	O1—H1F	0.8500
C2—N1	1.318 (4)		
			
O1—Cd1—Cl3	87.77 (5)	N1—C2—C1	127.5 (3)
O1—Cd1—Cl1	173.24 (5)	N2—C2—C1	126.8 (4)
Cl3—Cd1—Cl1	96.38 (3)	C4—C3—N2	106.3 (3)
O1—Cd1—Cl3^i^	87.31 (5)	C4—C3—H3	126.9
Cl3—Cd1—Cl3^i^	92.88 (3)	N2—C3—H3	126.9
Cl1—Cd1—Cl3^i^	97.78 (3)	C2—C1—H1A	109.5
O1—Cd1—Cl2^i^	81.00 (5)	C2—C1—H1B	109.5
Cl3—Cd1—Cl2^i^	168.67 (3)	H1A—C1—H1B	109.5
Cl1—Cd1—Cl2^i^	94.66 (2)	C2—C1—H1C	109.5
Cl3^i^—Cd1—Cl2^i^	88.14 (3)	H1A—C1—H1C	109.5
O1—Cd1—Cl2	84.77 (5)	H1B—C1—H1C	109.5
Cl3—Cd1—Cl2	87.60 (3)	C2—N1—C4	110.4 (3)
Cl1—Cd1—Cl2	90.06 (3)	C2—N1—H1D	124.8
Cl3^i^—Cd1—Cl2	172.04 (3)	C4—N1—H1D	124.8
Cl2^i^—Cd1—Cl2	89.85 (3)	C3—C4—N1	107.1 (4)
Cd1—Cl3—Cd1^ii^	93.11 (3)	C3—C4—H4	126.4
Cd1^ii^—Cl2—Cd1	90.42 (3)	N1—C4—H4	126.4
C2—N2—C3	110.5 (3)	Cd1—O1—H1G	110.9
C2—N2—H2A	124.8	Cd1—O1—H1F	110.4
C3—N2—H2A	124.8	H1G—O1—H1F	108.9
N1—C2—N2	105.7 (3)		
			
O1—Cd1—Cl3—Cd1^ii^	91.33 (5)	Cl2^i^—Cd1—Cl2—Cd1^ii^	−175.35 (4)
Cl1—Cd1—Cl3—Cd1^ii^	−83.31 (3)	C3—N2—C2—N1	0.7 (4)
Cl3^i^—Cd1—Cl3—Cd1^ii^	178.53 (4)	C3—N2—C2—C1	−179.2 (3)
Cl2^i^—Cd1—Cl3—Cd1^ii^	83.60 (12)	C2—N2—C3—C4	−0.4 (4)
Cl2—Cd1—Cl3—Cd1^ii^	6.48 (3)	N2—C2—N1—C4	−0.7 (4)
O1—Cd1—Cl2—Cd1^ii^	−94.37 (5)	C1—C2—N1—C4	179.1 (3)
Cl3—Cd1—Cl2—Cd1^ii^	−6.39 (2)	N2—C3—C4—N1	0.0 (4)
Cl1—Cd1—Cl2—Cd1^ii^	89.99 (3)	C2—N1—C4—C3	0.4 (4)
Cl3^i^—Cd1—Cl2—Cd1^ii^	−100.00 (16)		

Symmetry codes: (i) *x*, −*y*+1/2, *z*+1/2; (ii) *x*, −*y*+1/2, *z*−1/2.

### Hydrogen-bond geometry (Å, °)

**Table:**

*D*—H···*A*	*D*—H	H···*A*	*D*···*A*	*D*—H···*A*
N2—H2A···O1	0.86	2.13	2.884 (3)	146.
N1—H1D···Cl1^iii^	0.86	2.33	3.163 (3)	164.
O1—H1G···Cl1^i^	0.85	2.40	3.250 (2)	174.
O1—H1F···Cl1^iv^	0.85	2.44	3.174 (2)	146.

Symmetry codes: (iii) −*x*+2, *y*+1/2, −*z*+1/2; (i) *x*, −*y*+1/2, *z*+1/2; (iv) −*x*+1, *y*+1/2, −*z*+1/2.
